# A lower baseline glomerular filtration rate predicts high mortality and newly cerebrovascular accidents in acute ischemic stroke patients

**DOI:** 10.1097/MD.0000000000005868

**Published:** 2017-02-03

**Authors:** Kai Dong, Xiaoqin Huang, Qian Zhang, Zhipeng Yu, Jianping Ding, Haiqing Song

**Affiliations:** Department of Neurology, Xuanwu Hospital, Capital Medical University, Beijing, China.

**Keywords:** cerebrovascular disorders, chronic kidney disease, glomerular filtration rate, prognosis, stroke

## Abstract

Chronic kidney disease (CKD) is gradually recognized as an independent risk factor for cardiovascular and cardio-/cerebrovascular disease. This study aimed to examine the association of the estimated glomerular filtration rate (eGFR) and clinical outcomes at 3 months after the onset of ischemic stroke in a hospitalized Chinese population.

Totally, 972 patients with acute ischemic stroke were enrolled into this study. Modified of Diet in Renal Disease (MDRD) equations were used to calculate eGFR and define CKD. The site and degree of the stenosis were examined. Patients were followed-up for 3 months. Endpoint events included all-cause death and newly ischemic events. The multivariate logistic model was used to determine the association between renal dysfunction and patients’ outcomes.

Of all patients, 130 patients (13.4%) had reduced eGFR (<60 mL/min/1.73 m^2^), and 556 patients had a normal eGFR (≥90 mL/min/1.73 m^2^). A total of 694 patients suffered from cerebral artery stenosis, in which 293 patients only had intracranial artery stenosis (ICAS), 110 only with extracranial carotid atherosclerotic stenosis (ECAS), and 301 with both ICAS and ECAS. The patients with eGFR <60 mL/min/1.73m^2^ had a higher proportion of death and newly ischemic events compared with those with a relatively normal eGFR. Multivariate analysis revealed that a baseline eGFR <60 mL/min/1.73 m^2^ increased the risk of mortality by 3.089-fold and newly ischemic events by 4.067-fold. In further analysis, a reduced eGFR was associated with increased rates of mortality and newly events both in ICAS patients and ECAS patients. However, only an increased risk of newly events was found as the degree of renal function deteriorated in ICAS patients (odds ratio = 8.169, 95% confidence interval = 2.445–14.127).

A low baseline eGFR predicted a high mortality and newly ischemic events at 3 months in ischemic stroke patients. A low baseline eGFR was also a strong independent predictor for newly ischemic events in ICAS patients.

## 1 Introduction

Decreased renal function is an independent risk factor for all-cause and cardiovascular mortality in the general population, as well as in various populations with comorbidities.^[1–2]^ As chronic kidney disease (CKD) progresses, kidney-specific risk factors for cardiovascular events and disease come to play an important role.^[3]^ Previous study found that >50% of CKD patients died from a vascular disease, including stroke.^[4]^ However, the silent nature of the kidney often leads medicine to overlook CKD. In addition, it also loses the opportunity to prevent kidney and vascular diseases.^[5]^ CKD has a higher prevalence among patients with cardiovascular diseases and is increasingly considered as an independent risk factor for cardiovascular disease and stroke.^[6–7]^ However, the impact of low estimated glomerular filtration rate (eGFR) on the clinical outcomes of acute ischemic stroke remains controversial, including its effect on all-cause mortality, newly cerebrovascular accidents, and functional disabilities.^[6,8]^

Stroke is the leading cause of disability and the third leading cause of mortality in the world. The burden of stroke in China is likely the highest in the world, affecting 2.5 million people each year.^[9]^ According to TOAST criteria, cerebrovascular (carotid artery and/or intracranial artery) stenosis led by atherosclerosis is the most common cause of ischemic stroke.^[10]^ In addition, intracranial artery stenosis (ICAS) has a higher prevalence in Asian ischemic stroke patients in contrast to a higher prevalence of extracranial carotid atherosclerotic stenosis (ECAS) in white stroke patients.^[11]^ The higher prevalence of intracranial artery stenosis may partly explain the huge burden of ischemic stroke in China. In addition, patients with ICAS have a more severe stroke and a higher risk of recurrence.^[12]^ However, previous studies paid less attention to potential role of kidney dysfunction on clinical outcomes of ischemic stroke patients with ICAS.

Therefore, this study was designed to investigate the effect of baseline eGFR on clinical outcomes at 3 months following the onset of ischemic stroke. In addition, the impact of CKD on clinical outcomes was further analyzed in patients with ICAS and ECAS.

## 2 Material and methods

### 2.1 Patients

This investigation was a retrospective study, which was approved by the Ethics Committee and Institutional Review Board of Xuan Wu Hospital. Written informed consent to participate was obtained from each subject or his/her families after receiving a full explanation for the purpose and nature of the study.

All patients were diagnosed as having ischemic stroke according to American Heart Association/American Stroke Association recommendations and were further confirmed by CT or MRI scan in the hospital.^[12–13]^ The inclusion criterion was patients (older than 18 years) with an acute ischemic stroke within 7 days after the onset. A total of 1146 patients with acute cerebral infarction were recruited into data analysis from August 2011 to August 2014. Exclusion criteria included complications of other brain illness (e.g., brain tumor, hypnotic-ischemic encephalopathy), dementia, undergoing dialysis, and incomplete data at baseline or at follow-up. Finally, a total of 972 patients were included into this study.

### 2.2 Clinic data

Details of patient demography, risk factors, laboratory, and brain image data were recorded. General information on patients was collected, including age, sex, body mass index (BMI), history of hypertension, diabetes, dyslipidemia, smoking, and alcohol consumption. The results of laboratory tests were also collected, including serum lipids, fasting glucose, blood routine test, blood coagulation function. Hypertension (previous diagnosis, antihypertensive treatment, or blood pressure >140/90 mmHg on at least 2 measurements at different time points), diabetes mellitus (previous diagnosis, concurrent treatment with insulin or oral hypoglycemic medications, or fasting plasma glucose level >7.0 mmol/L or random blood glucose level >11.1 mmol/L), hypercholesterolemia (self-reported history of hypercholesterolemia, lipid-lowering therapy, cholesterol >5.72 mmol/L), coronary heart disease (myocardial infarction and stable angina), status of smoking (smoking >10 cigarettes a day before onset, for at least 6 months), and alcohol (the average daily drinking >50 g for >1 year), were diagnosed according to generally accepted criterion.^[6,14–15]^

### 2.3 Determination of cerebral artery stenosis

Cerebral artery stenosis was determined by at least 2 methods: vascular ultrasound, magnetic resonance angiography (MRA), and computed tomographic angiography (CTA). When atherosclerotic lesions were found in the cerebral arteries, the maximum percentage of stenosis was calculated based on the residual lumen diameter and distance from the original walls at the site of maximum lumen narrowing.^[16]^ The cerebral artery stenosis was classified as 5 types: normal (without stenosis), mild stenosis (1% to 49%), moderate stenosis (50% to 69%), severe stenosis (70%–99%), and total occlusion (>99%). When there was agreement between 2 methods, the type of cerebral artery stenosis was determined. A third method (MRA or CTA) was adopted when disagreement occurred. When multiple cerebrovascular stenoses existed, the severest stenosis was taken into account.^[16–18]^ The anatomical range of involved blood vessels were classified as normal, intracranial artery stenosis (C2–C7 segments of internal carotid artery, anterior cerebral artery, middle cerebral artery, posterior cerebral artery, basilar artery, and V5 segment of vertebral artery), extracranial artery stenosis (common carotid artery, C1 segment of internal carotid artery, and vertebral artery before V5 segment), intra- and extracranial artery stenosis.

### 2.4 Estimation of GFR and CKD

eGFR was assessed using the following equation from the Modification of Diet in Renal Disease. The formula was as follows: eGFR (mL/min/1.73m^2^) = 175 × Scr-1 234 (mg/dL) × age-0 179 (×0.79 if female). Serum creatinine level was evaluated within 24 hours after admission. Patients were divided into 3 groups according to the eGFR: CKD phase 1 group, including patients with an eGFR ≥90 mL/min/1.73m^2^; CKD phase 3–5 group, including patients with an eGFR <60 mL/min/1.73 m^2^; and CKD phase 2 group, including patients with eGFR between 60 and 90 mL/min/1.73m^2^. CKD phase 1–2 is regarded as normal function and as non-CKD group, and CKD phase 3–5 are considered as renal dysfunction and as CKD group.^[1,19–20]^

### 2.5 Endpoint analysis

Patients were followed-up for 3 months after the onset of ischemia. Phone follow-up interviews or outpatient interviews were conducted by professional interviewers. Endpoint events included all-cause death and newly ischemic events, including ischemic stroke and TIA. The diagnosis of cerebrovascular events was based on the American Heart Association/American Stroke Association recommendations.^[12–13]^

### 2.6 Statistical analysis

Statistical analysis was performed using SPSS software, version 20.0 (SPSS Inc, Chicago, IL). Measurement data were expressed as means + SD. Categorical variables were expressed as percentage. Comparison among groups was tested by 1-way analysis of variance or *χ*^2^ analysis. Intergroup differences in risk were assessed using the log-rank test. Patients with an eGFR of ≥90 mL/min/1.73 m^2^ served as the reference group. Independent factors that were significantly related to a poor functional outcome were identified using a multivariate model, with adjustments being made for variables with *P* < 0.05 in univariate analysis.

## 3 Results

### 3.1 Baseline characteristics of patients

Totally, 972 ischemic stroke patients (517 males, 455 females) were included, in which 130 patients (13.4%) had reduced eGFR (<60 mL/min/1.73 m^2^), 556 patients had a normal eGFR (≥90 mL/min/1.73 m^2^), 286 patients with a relatively normal eGFR (between 60 and 90 mL/min/1.73m^2^). There were 90 patients in phase 3 CKD and 40 patients in phase 4 CKD. Of all patients, 278 cases did not have cerebral artery stenosis, and 694 suffered from cerebral artery stenosis, in which 293 patients had ICAS, 110 had ECAS, and 301 had both ICAS and ECAS. Baseline parameters were shown in Table 1. Patients with lower eGFR were older, more likely to be women, frequently with a higher National Institute of Health stroke scale (NIHSS) scores. However, there were no significant differences in stenosis degree and site among three groups.

**Table 1:**
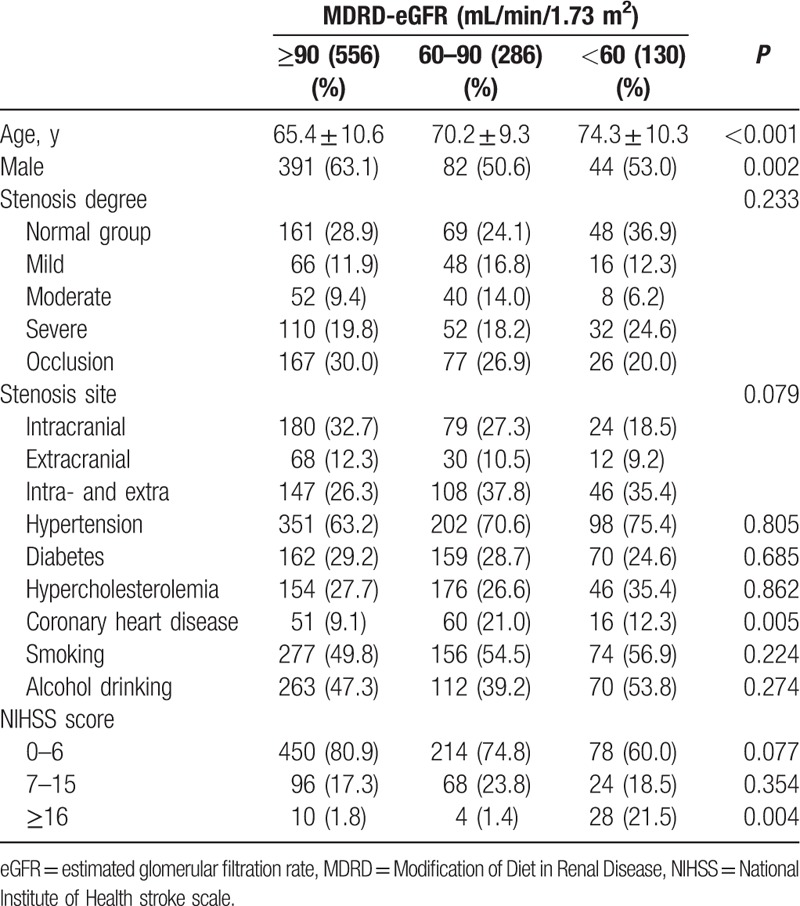
Baseline characteristics by eGFR.

### 3.2 Clinical outcomes at the end of follow-up

At the end of follow-up, 29 patients died and 79 suffered from newly ischemic events. The patients with eGFR <60 mL/min/1.73 m^2^ had a higher proportion of death and newly events compared with those with a relatively normal eGFR (Table 2). The patients with renal dysfunction had a higher risk of death (odds ratio [OR] = 3.089, 95% confidence interval [CI] = 1.141–6.093) and newly ischemic events (OR = 4.067, 95% CI = 2.431–8.633).

**Table 2:**
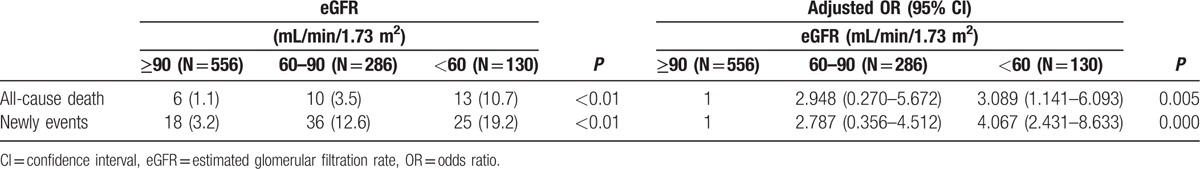
Clinical Outcomes by eGFR for all patients at 3 months.

### 3.3 Clinical outcomes for patients with ICAS and ECAS

At the end of follow-up period, 8 ICAS patients died and 18 suffered from newly ischemic events. Four ECAS patients died and 6 suffered from newly ischemic events. Reduced eGFR was associated with increased rates of mortality and newly events both in ICAS patients and ECAS patients (Table 3). However, only an increased risk of newly events was found as the degree of renal function deteriorated in ICAS patients (OR = 8.169, 95% CI = 2.445–14.127) (Table 4).

**Table 3:**
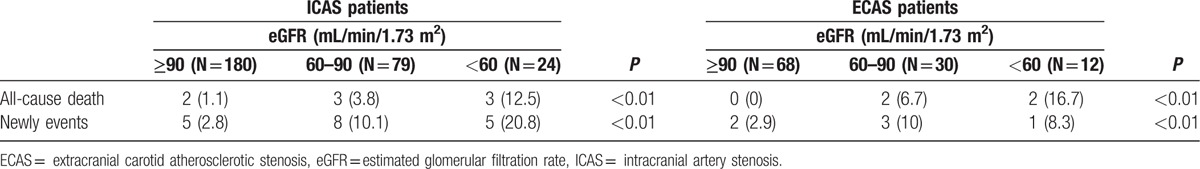
Clinical outcomes by eGFR for patients with ICAS and ECAS at 3 months.

**Table 4:**
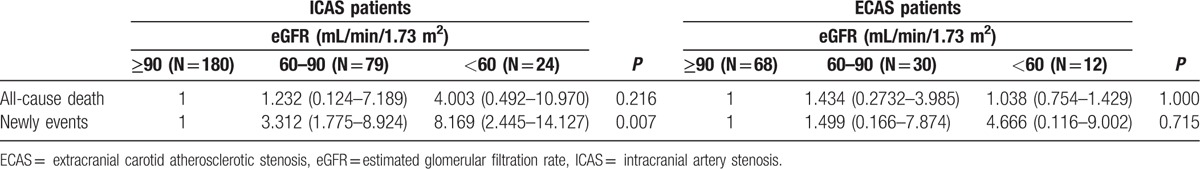
Logistic regression models for prediction of outcomes by eGFR for patients at 3 months.

## 4 Discussion

The incidence and morbidity of cerebrovascular disease and chronic kidney disease are increasing in both China and worldwide. The relationship between renal dysfunction in various degree and increased cerebrovascular morbidity and mortality is widely accepted.^[10,21]^ As previously reported, we also found that a reduced eGFR predicted an increased rate of mortality and newly ischemic events in ischemic stroke patients. Our main finding was that a lower eGFR was an independent predictor of newly ischemic events in ischemic stroke patients with ICAS in further analysis.

Patients with CKD had a significant higher morbidity and mortality because of cardio/cerebral vascular disease than normal patients.^[1,22]^ Approximately 14% to 16% Chinese stroke patients had renal malfunction of various degree based on previous report.^[23]^ In addition, approximately 50% stroke patients suffered from ICAS in China, which were associated with a severe stroke, a longer period in the hospital, and a higher risk of recurrent stroke.^[12]^ Various outcomes had been presented in researches concerning CKD phases and the prognosis of ischemic stroke.^[6,24–27]^ Some studies revealed that an impaired kidney function was related with a poor long-term prognosis.^[24–26]^ In contrast, other studies found that reduced eGFR was not an independent predictor of death/disability at the end of the 12th month in patients with ischemic stroke.^[6,27]^ However, less study made a further analysis on the association between a low eGFR and the prognosis of ICAS and ECAS patients. In this study, we only found that a decreased eGFR could increase relative odds for mortality in ischemic stroke patients, but we did not find increased relative odds for mortality in ICAS or ECAS patients. Traditional risk factors for vascular disease and stroke, including but not limited to hypertension, lipid abnormalities, and diabetes, participated in the progress of kidney dysfunction, whereas CKD also promoted high blood pressure and lipid abnormalities.^[6,28]^ So non-neurological organ dysfunction could worsen neurological impairment and contribute to an increased risk of mortality. In the further analysis, we only included patients with ICAS or ECAS without patients with both ICAS and ECAS. So it was possible that these patients did not suffer from a severe neurological impairment and non-neurological organ dysfunction. In mechanism, the presence of renal dysfunction and other risk factors could result in the increased concentration of asymmetric dimethylarginine inhibiting generation of nitric oxide, the low-grade inflammation raising oxidative stress, dyslipidaemia, and the elevated activity of the renin–angiotensin system stimulating production of superoxide and cytokines.^[28–29]^ Thus, the progression of endothelial dysfunction and following atherosclerosis was probably one of the mechanisms for stroke in patients with reduced eGFR.

We only found that a lower eGFR was an independent predictor of newly ischemic events in ischemic stroke patients with ICAS. There were obvious anatomical differences between ICAS and ECAS. The histological features and stroke mechanisms of ICAS atherosclerosis also differed from those of ECAS atherosclerosis.^[30]^ Previous studies found that hypertension and diabetes mellitus were significant risk factors for ICAS compared with ECAS, whereas hyperlipidemia and smoking were more closely associated with ECAS compared with ICAS.^[31]^ In the analysis of ischemic stroke mechanism, ICAS was prone to lead to internal border-zone infarcts, whereas ECAS was prone to result in artery-to-artery embolism.^[32]^ In prognosis analysis, patients with ICAS had a higher risk of recurrent stroke than patients with ECAS.^[32]^ In another study, patients with ICAS had poor long-term prognoses (a higher morbidity and mortality rate) under the conditions of current medical therapy.^[33]^ Previous study observed that reduced eGFR was closely associated with carotid atherosclerosis in patients with CKD.^[34]^ Recent studies also discovered that CKD and CKD progression were independently associated with presence and evolution of deep or infratentorial cerebral microbleeds. Renal and brain had similar anatomical and functional features of supplying arteries.^[35]^ They also shared similar dynamic changes because of susceptibilities to vascular damage.^[36–37]^ The anatomical, histological, and pathological features of ICAS may be the basis for the association of a lower eGFR and a higher newly ischemic event.

Our research has several limitations. First, this study was an observational study, not a randomized controlled trial. Secondly, the sample size was small, especially when we made a further analysis in ICAS and ECAS patients, only collecting ischemic stroke patients from one hospital. Thirdly, we only made a short-term follow-up for 3 months. A long-term observation may give more information concerning the effect of the kidney dysfunction on cerebrovascular disease. In addition, we just evaluated kidney function with Modification of Diet in Renal Disease formula. Although it is generally accepted as a valid surrogate of kidney function, additional measurement of albuminuria (albumin to creatinine ratio) and cystatin-C may improve our assessment of CKD. In further analysis, more acute ischemic stroke patients, including ICAS, ECAS, and both, would be included to analyze the predictive ability of CKD for acute ischemic stroke.

## 5 Conclusions

This study demonstrated that a lower eGFR predicted a high mortality and newly ischemic events at 3 months in ischemic stroke patients. In particular, a low baseline eGFR also predicted a higher newly ischemic event in ICAS patients.
